# Ubiquitin-Mediated Control of ETS Transcription Factors: Roles in Cancer and Development

**DOI:** 10.3390/ijms22105119

**Published:** 2021-05-12

**Authors:** Charles Ducker, Peter E. Shaw

**Affiliations:** Queen’s Medical Centre, School of Life Sciences, University of Nottingham, Nottingham NG7 2UH, UK

**Keywords:** E3 ligase complex, deubiquitinase, gene fusions, mitogens, phosphorylation, DNA damage

## Abstract

Genome expansion, whole genome and gene duplication events during metazoan evolution produced an extensive family of *ETS* genes whose members express transcription factors with a conserved winged helix-turn-helix DNA-binding domain. Unravelling their biological roles has proved challenging with functional redundancy manifest in overlapping expression patterns, a common consensus DNA-binding motif and responsiveness to mitogen-activated protein kinase signalling. Key determinants of the cellular repertoire of ETS proteins are their stability and turnover, controlled largely by the actions of selective E3 ubiquitin ligases and deubiquitinases. Here we discuss the known relationships between ETS proteins and enzymes that determine their ubiquitin status, their integration with other developmental signal transduction pathways and how suppression of ETS protein ubiquitination contributes to the malignant cell phenotype in multiple cancers.

## 1. Introduction

Cell growth, proliferation and differentiation are complex, concerted processes that rely on careful regulation of gene expression. Control over gene expression is maintained through signalling pathways that respond to external cellular stimuli, such as growth factors, cytokines and chemokines, that invoke expression profiles commensurate with diverse cellular outcomes. Downstream effectors of these signalling pathways are transcription factors, a generic term used to describe a broad range of proteins that coordinate gene expression patterns by either activating or repressing specific target genes. They achieve this through recognition of DNA and protein binding motifs, which vary between transcription factor families; they also recruit additional proteins to aid transcriptional repression or activation (known as co-repressors and co-activators respectively) [1].

Members of the E-twenty-six/E26 (ETS) family of transcription factors all share a conserved DNA-binding domain with a winged helix-turn-helix structure that recognises purine-rich DNA sequences with a GGA core [2]. *ETS* genes were originally discovered through the viral ets (*v-ets*) oncogene within the leukaemia-causing avian retrovirus E26, which was found to have been transduced from homologous genes in the chicken genome to encode part of a hybrid viral protein [3]. This led to the discovery of human *ETS* genes (*ETS-1*, *ETS-2*, and later *ERG*) and the proteins encoded by these genes [4]. In the human genome 28 genes encoding ETS-transcription factors have been identified and divided into various sub-families (ETS, ERG, ELG, TEL, PEA3, ETV2, PDEF, ESE, ELF, SPI, ERF and TCF) with each sub-family comprising between 1–3 members [5]. They can act as transcriptional activators or repressors to regulate gene targets, and can vary substantially outside of the ETS domain [6]. In addition, several sub-families also harbour a conserved helix-loop-helix (HLH) or pointed (PNT) domain (also known as sterile alpha motif [SAM]), which is involved in mediating protein-protein interactions [7].

The majority of ETS proteins are phosphorylation targets of mitogen-activated protein kinases (MAPKs) including extracellular signal-regulated kinases (ERKs), C-JUN N-terminal kinases (JNKs) and p38 kinases [8]. Several are key ERK responders, and as such are involved in the regulation of a diverse portfolio of cellular processes including proliferation, differentiation and survival [9,10,11]. As such, dysregulated expression and activities of ETS family members are frequently reported in cancers, with oncogenic fusions also prevalent [5,12,13]. Aside from phosphorylation, ETS proteins are regulated by other post-translational modifications, including acetylation, SUMOylation and the focus of this review, ubiquitination [14,15].

Ubiquitination involves conjugation of the C-terminal glycine from the 8.6 KDa protein ubiquitin to protein targets, most commonly through lysine moieties, although serine, threonine, cysteine and N-terminal methionine residues can also be modified [16]. This occurs through an ATP-driven enzymatic cascade involving E1 ubiquitin-activating, E2 ubiquitin-conjugating and E3 ligase enzymes, of which there are 2, ~40 and 600–700 respectively in humans, and importantly, can be reversed by deubiquitinases (DUBs), of which there are ~100 [17,18,19,20]. The seven internal lysines within ubiquitin and the N-terminal methionine can also serve as ubiquitin acceptors, giving rise to di/tri/polyubiquitin chain formation and extension. K48-linked chains are classically associated with promoting target degradation through the ubiquitin proteasome system (UPS), but the variety of potential modification types and chain topologies affords significant signalling complexity, altering the interaction landscape and conferring various possible outcomes on client proteins [21,22].

Tight regulation of protein degradation and recycling is crucial in maintaining cellular homeostasis, and dysregulated turnover of oncoproteins and tumour suppressors (TS) is a common hallmark of cancer, marking the proponents of ubiquitin conjugation and removal as targets for potential therapeutic intervention [23,24]. This review focusses on the role ubiquitination plays in regulating ETS transcription factor function and its impact on developmental processes and oncogenesis.

## 2. ETS-1, ETS-2 and the Constitutive Photomorphogenesis 1 (COP1) Complex

The founding member of the ETS transcription family ETS-1 shows extensive sequence conservation among vertebrates and alongside its sibling ETS-2 has critical roles in the development of the heart and circulatory system [25,26,27]. In adult humans, mice and chickens, ETS-1 expression is particularly localized to immune tissues (e.g., thymus, spleen and lymph nodes in mice), consistent with roles in regulating B cell, T cell and NK cell differentiation, whereas ETS-2 expression is more widespread [11,25,28].

**Figure 1:**
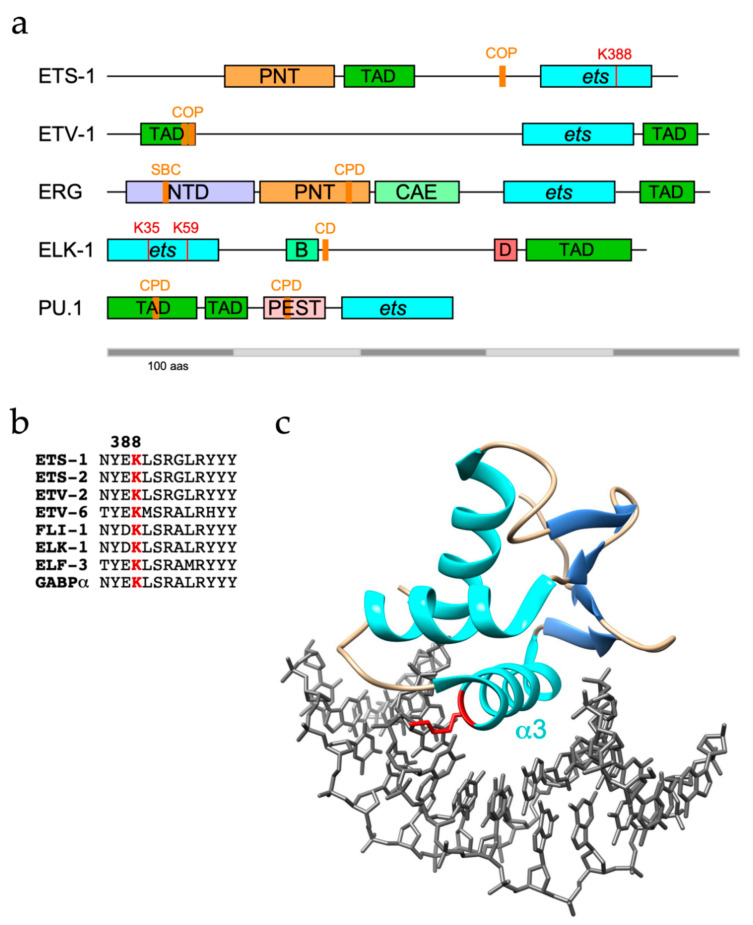
(**a**) Domain organisation of ETS proteins with established ubiquitination modalities indicating ubiquitinated lysines confirmed by a combination of mutagenesis and MS in red and characterised degron motifs as orange bars: COP, constitutive photomorphogenesis 1 degron; SBC, SPOP-binding consensus; CPD, CDC4 phospho-degron; CD, cryptic degron. Domains are indicated thus: PNT, pointed (peach); TAD, transactivation (green); ets, ETS (cyan); NTD, N-terminal (lilac); CAE, central alternative exons (light green); B; SRF-interaction (sea green); PEST, proline/glutamate/serine/threonine-rich (pink). (**b**) Alignment of ETS domain α3 helices indicating conservation (in 25/28 human ETS proteins) of most frequently modified lysine in red, as determined by MS, including large-scale proteomic analysis. See Table 2 for references. (**c**) ETS domain of ELK-1 (PDB: 1DUX) bound to consensus ETS binding site, showing alpha-helices in cyan and beta-strands in blue with α3 inserted in major groove and sidechain of K59 (K388 in ETS-1) in red making contact with core GGA sequence [33].

ETS-1 and ETS-2 are both targets for ubiquitination, with ETS-1 found to be modified with K48-linked polyubiquitin chains and targeted to the proteasome [29]. However, as well as confirming ETS-1 as a UPS substrate, another study identified K63-linkages as the major ETS-1 polyubiquitin topology, which are generally, but not exclusively, associated with proteasome-independent pathways [30,31]. The C-terminal ETS domain of ETS-1 appears to be the region targeted for modification (Figure 1a), with K388 identified as an ubiquitination site by diglycine remnant mapping, a mass spectrometry (MS)-based method for ubiquitination site identification, and confirmed by mutational analysis [30,32].

**Figure 2:**
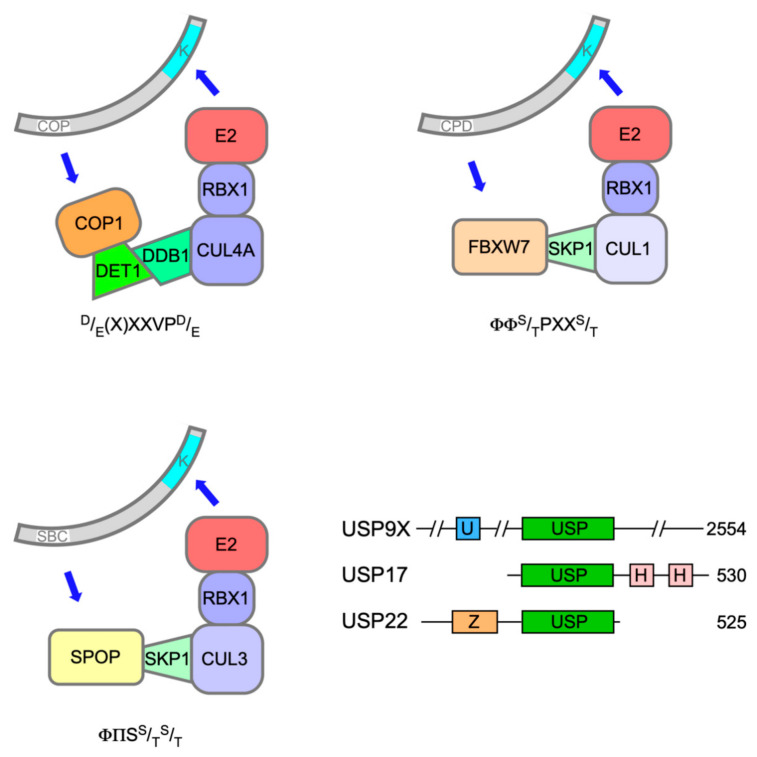
Subunit composition of E3 ligase complexes and domain structures of DUBs implicated in regulation of ETS protein activity. Each E3 ligase recognition subunit recruits its targets by means of a short consensus motif, indicated below each complex (Ф = hydrophobic; П = hydrophilic), to allow transfer of ubiquitin from the bound E2 activating enzyme to one or more target lysines, predominantly within the ETS domain. Target phosphorylation within the consensus motif may be a prerequisite or an adjunct for binding, whereas phosphorylation of residues adjacent to the motif can inhibit binding (see text for details). DUBs implicated in ETS protein ubiquitination all share a conserved USP domain (green) with a catalytic triad of cysteine, histidine and aspartic acid residues (two in the case of USP9X), but few other recognised features in common. U, ubiquitin-like (UBL) domain (blue); H, hyaluranon binding motif (pink); Z, Zinc finger (peach). Numbers to the right indicate protein size in amino acids.

Polyubiquitination of both ETS-1 and ETS-2 is catalysed by Constitutive Photomorphogenesis 1 (COP1, also known as RFWD2), a really interesting new gene (RING) E3 ligase, which, through its adapter protein binding partner De-etiolated 1 (DET1), can complex with Cullin 4a (CUL4a), damage-specific DNA binding protein 1 (DDB1) and RING-box protein 1 (ROC1) (Figure 2) to mediate target ubiquitination [34,35]. The expression of dominant-negative Cullins had previously flagged ETS-2 as a substrate of Cullin-RING Ligase 4 [36].

COP1 activity on ETS-1 and ETS-2, prompting their subsequent degradation, was triggered by phosphorylation within atypical COP1 degrons [classically (D/E)-(D/E)-(X)-X-X-V-P-(D/E)], in which serine or threonine replaced aspartic/glutamic acid and their phosphorylation provided the negative charge required for COP1 docking (one degron in ETS-1, two in ETS-2) [35]. A relevant phosphorylation event in an ETS-2 degron (S310) mediated by Ca^2+^/calmodulin-dependent kinase 2 (CaMKII) had previously been reported [37].

Phosphorylation by SRC kinases of a degron-adjacent tyrosine residue in ETS-1 (Y293), which is absent in ETS-2 degrons, abrogated COP1 binding, revealing antagonistic roles for phosphorylation in the regulation of ETS-1 ubiquitination [35]. SRC-mediated escape of ETS-1 from COP1 has implications in triple-negative breast cancer (TNBC), where a significant correlation was found in tissue samples between SRC phosphorylation and ETS-1 protein levels. An increase in SRC activity in MCF10A immortalised breast epithelial cells also led to increased ETS-1 levels and promoted anchorage-independent growth [35]. It should be noted that the SUMO E3 ligase protein inhibitor of activated STAT4 (PIAS4) was shown to bind to the transactivation domain (TAD) of ETS-1 and prevent its turnover by the proteasome, albeit without affecting ETS-1 ubiquitination status [38].

ETS-2 was found to be phosphorylated by cyclin-dependent kinase 10 (CDK10)-Cyclin M, which stimulated its degradation, highlighting the region around S220 and S225, the major phosphorylation sites, as a probable phosphodegron [39]. Alanine substitutions at S220, S225 and S248 inhibited the interaction of DET1 with ETS-2, which is required to promote COP1-mediated proteasomal degradation, suggesting that phosphorylation of these sites by CDK10 primes ETS-2 for ubiquitination [40]. An oncogenic mutant of p53 (mtp53) was able to prevent ETS-2 turnover by competing with DET1 for binding to the CDK10-targeted region and having a destabilising effect on DET1 [40,41]. It is also worth noting that ETS-2 is stabilised in mouse embryonic fibroblasts deficient in the E3 ligase anaphase promoting complex (APC) subunit cadherin 1 (Cdh1), suggesting there are likely to be multiple routes for ETS-2 turnover through the UPS [42].

One enzyme responsible for the removal of polyubiquitin chains from ETS-1 has been identified. Ubiquitin-specific protease USP9 X-linked, originally identified in the context of developmental signalling, was found to be a *bona fide* ETS-1 DUB [43]. USP9X was shown to interact directly with ETS-1 and prevent its proteasomal degradation through deubiquitination, promoting ETS-1 transcriptional activity at the *NRAS* promoter in melanoma cells [30]. NRAS mutant melanoma cell growth and survival relies on continual *NRAS* expression, implying that USP9X-mediated stabilisation of ETS-1 drives tumorigenesis. Accordingly, USP9X depletion in melanoma cell-derived tumours abrogated growth in mouse xenograft models, while its over-expression significantly increased tumour expansion [30]. USP9X effects on ETS-1 stability appear not to extend to ETS-2, because USP9X knockdown in melanoma cells decreased ETS-1 protein levels but left ETS-2 unaffected [30].

In summary, polyubiquitination of ETS-1 and ETS-2 involves the same E3 ubiquitin ligase complex, COP1, but their modification is regulated by different kinases and its reversal may involve distinct DUBs. This regulatory divergence presages the arcane interrelationships between the ubiquitination machinery and other members of the ETS protein family.

## 3. ERG Fusion Proteins and the Evasion of Ubiquitin-Mediated Proteolysis in Prostate Cancer

ERG and its close relative within the *ETS* gene family, *FLI-1* (friend leukaemia integration 1), both participate in regulating haematopoietic stem cell development [44,45]. However, ubiquitin-mediated control of ERG has been charted largely in the context of prostate cancer (PCa). Gene rearrangements involving *TMPRSS2* (transmembrane protease serine 2) and one of several *ETS* genes is a frequent occurrence in PCa [46]. The *TMPRSS2* gene is androgen responsive and juxtaposition of the *TMPRSS2* 5′-UTR upstream of ETS coding sequences upregulates their expression. *TMPRSS2:ETS* fusions most frequently involve *ERG* (40%) but also *ETV1*, *ETV4* and *ETV5* (see Section 4 below). Fusion-mediated over-expression of ERG proteins with short N-terminal deletions results in the up-regulation of ERG target genes commensurate with cell proliferation and migration. In advanced castration-resistant prostate cancer (CRPC), *TMPRSS2:ERG* fusions often accompany lesions in TS genes, notably *PTEN* (phosphatase and tensin homologue).

The loss or reversal of ubiquitination contributes significantly towards increased ERG levels and activity in PCa. The first component of this regulatory plexus to be identified was USP9X, the aforementioned DUB for ETS-1. USP9X was shown to deubiquitinate ERG and promote its stability in VCaP cells [47]. Depletion or inhibition of USP9X led to reduced ERG expression that was linked to impaired PCa signature gene expression and the inhibition of ERG-positive tumour growth in mouse xenograft models.

The impact of USP9X inactivation on ERG stability implied that its levels were tightly controlled by the UPS. In support of this notion, several E3 ubiquitin ligases for ERG have been characterised. The first to be discovered was the tripartite motif-containing TRIM25, an E3 ligase implicated in innate immune responses [48]. TRIM25 was shown to polyubiquitinate ERG in cells and in vitro [49]. ERG up-regulates TRIM25 expression in PCa cells, suggesting the existence of a negative feedback loop, but the action of USP9X minimises this effect by deubiquitinating and stabilising ERG. Of note, TRIM25 is able to ubiquitinate PCa-specific, N-terminal truncations of ERG [49].

The speckle-type POZ (pox virus and zinc finger) protein (SPOP) is a substrate adaptor for CUL3-RING E3 ubiquitin ligases (Figure 2) [50]. The *SPOP* gene was identified as the most frequent target of somatic substitutions in PCa [51]. Mutations affecting key residues in the substrate recognition (MATH) domain of SPOP were observed in 6–15% of PCa tumours. Such mutant SPOP proteins fail to recognise their targets, which include androgen receptor (AR) [52].

SPOP targets ERG for destruction through recognition of a SPOP binding consensus (SBC) located towards the N-terminus of ERG (Figure 1a) and TMPRSS2-ERG fusions (Δ39 and Δ99) were shown to evade SPOP due to disruption or deletion of the SBC [52,53]. Nonetheless, an ERG mutant lacking the SBC and unable to bind SPOP was still degraded, consistent with the existence of other E3 ligases for ERG [52].

Phosphorylation of serine(s) in the SBC may aid substrate recognition by SPOP and casein kinase 1δ (CK1δ) has been implicated in phosphorylation of ERG within the SBC. Treatment of VCaP cells with DNA damaging drugs such as etoposide or doxorubicin stimulated CK1δ activity (nuclear localisation) and promoted ERG degradation [53].

It had been noted that *SPOP* mutations do not co-associate with *TMPRSS2-ERG* fusions common in PCa [51]. However, gene expression signatures of PCa with *SPOP* mutations and *TMPRSS2:ERG* fusions appeared to be similar, leading to the inference that increased ERG activity was a main oncogenic driver in cancers with *SPOP* mutations [52,53]. But a subsequent study of PCa in mouse models and patient samples found no association between the presence of inactivating *SPOP* mutations and ERG expression [54]. Moreover, the gene expression signatures shared between *SPOP* mutation and *TMPRSS2-ERG* fusion cancers consisted largely of genes expressed in normal prostate tissue. Thus it appears that *ERG* rearrangements and *SPOP* mutations characterise distinct forms of PCa [55].

Recent evidence has implicated a third E3 ubiquitin ligase in the destruction of ERG and *TMPRSS2:ERG* fusion derivatives in response to DNA damage. F-box and WD40 repeat domain containing 7 protein (FBXW7/CDC4) is the substrate recognition subunit of an S phase kinase-associated protein 1 (SKP1)-CUL1-F-box (SCF) E3 ligase complex (Figure 2) and has been ascribed significant TS properties [56]. Treatment of PCa cells with camptothecin or ionising radiation decreased ERG protein levels commensurate with an increase in their ubiquitination catalysed by FBXW7 [57].

FBXW7 recognises its targets via a characteristic phosphodegron (I/L/P-T*-P-x-x-S*/T*, where * denotes phosphate). Phosphorylation of the N-terminal threonine is usually accomplished by GSK-3β but requires priming phosphorylation of the C-terminal serine/threonine residue (or D/E phosphomimetic substitution). ERG proteins were found to harbour an atypical phosphodegron (^186^L-T-P-S-Y^190^) that was recognised by FBXW7, in which GSK-3β phosphorylation of T187 was primed by phosphorylation of Y190. Screening components of DNA damage response pathways identified WEE1 as the probable Y190 priming kinase [57].

Inhibitory phosphorylation of GSK-3β by protein kinase B (PKB/AKT) as a consequence of mitogenic growth factor signalling serves to prevent FBXW7-mediated ERG destruction but is normally countermanded by the phosphatase activity of PTEN [58]. Hence *PTEN* loss, a frequent occurrence in CRPC, may increase ERG protein stability and potentially confer resistance to genotoxic therapy [57].

The *FLI-1* gene has a similar propensity for rearrangement and is implicated in the aetiology of approx. 85% of cases of Ewing Sarcoma, a childhood bone malignancy [59]. Tumour cells characteristically express fusion proteins with an N-terminal region derived from EWS, a poorly characterised RNA-binding protein, and C-terminal region from FLI-1, or less frequently ERG, including the ETS domain. EWS-FLI-1 fusions are short-lived proteins that become ubiquitinated in the ETS domain and turned over by the UPS, although the E3 ubiquitin ligase responsible remains to be identified [60]. Of note, the same lysine (K334: = K388 in ETS-1) was also ubiquitinated on FLI-1. However, neither SBC nor CPD motif present in ERG is conserved in FLI-1, implying that it is unlikely to be a substrate for SPOP or FBXW7. More recently, USP19 was shown to deubiquitinate and stabilise EWS-FLI-1 fusions but not FLI-1, likely because USP19 bound to EWS-FLI-1 within N-terminal EWS sequences [61]. The observed modest impact of *USP9X* depletion on levels of EWS-FLI-1 hints that the role of USP9X may be conserved between ERG and FLI-1 [61].

Mechanisms that govern ERG function appear to be shared more broadly among ETS proteins. PU.1 (or SPI-1, of the SPI subfamily) plays an essential role in lineage commitment of haematopoietic precursor cells into macrophages and monocytes whereby impaired PU.1 function has been linked to the development of acute myeloid leukaemia (AML) [62]. In common with ERG, PU.1 contains two phosphodegrons that can be phosphorylated by GSK-3β and subsequently bound by FBXW7, leading to its ubiquitination and degradation (Figure 1a) [63]. Blockade of the GSK-3β-FBXW7 signalling axis restored PU.1 levels in peripheral blood mononuclear cells and promoted their differentiation. Related to these findings is an earlier report that in combination with an oncogenic *Kras* allele, loss of USP22, a component of the SPT-ADA-GCN5 acetyltransferase (SAGA) complex, promoted AML [64]. USP22 was shown to deubiquitinate and protect PU.1 from degradation in Kras-positive myeloid progenitors and thus promote their differentiation [65].

These studies illustrate that ERG, its close relatives and their fusion derivatives are substrates for multiple E3 ligase complexes, subject to the availability of functional phosphodegrons, but conserved lysines within the ETS domain appear to be the preferred sites for ubiquitin conjugation. How the different E3 ligase complexes for ERG impact on haematopoiesis remain to be established.

## 4. Interplay between PEA3 Relatives and COP1 during Insulin Secretion and in Cancer

The proto-oncogenic polyoma enhancer activator protein (PEA3, also known as ETS variant 4 [ETV4]/E1AF), alongside ETV1 (ER81) and ETV5 (ERM) make up the PEA3 subfamily of ETS-domain transcription factors. PEA3 members are associated with organogenesis, having roles in kidney, mammary gland, limb and lung development, with some redundancy apparent between them when expressed within the same tissue [66,67].

All PEA3 members are subject to control by the UPS. Initially, ETV-4 had been shown to be ubiquitinated within a C-terminal region encompassing the ETS domain and stabilised by proteasomal inhibition [68]. Ubiquitination and turnover of ETV4 appeared to be promoted by SUMOylation at multiple sites in the N-terminal region, which was enhanced by ERK cascade stimulation [69]. However, a later study found that treatment of HCT-116 colorectal cancer cells with MEK inhibitor U0126 promoted ETV4 ubiquitination and degradation [70]. ETV5 was also found to be ubiquitinated and degraded, although the major modified species of ETV5 appeared to be mono-ubiquitinated with some polyubiquitination apparent following proteasomal inhibition [71].

As with ETS-1 and ETS-2, COP1 also polyubiquitinates ETV1, ETV4 and ETV5 in partnership with DET1 [72,73]. COP1-mediated destruction of ETV1/4/5 is mainly achieved through two adjacent conventional COP1 degrons residing within the N-terminal TADs that are conserved across PEA3 family members (Figure 1a) [72,73]. Phosphorylation of ETV4 by ERK on S73 in between these in-tandem degrons (this site is absent in ETV1 and ETV5) in HCT-116 cells prevented COP1 interactions and stabilised ETV4 [70].

Mirroring the phenomenon described for ERG, truncated ETV1 proteins expressed in PCa from *TMPRSS2:ETV1* fusions lack COP1 phosphodegrons and hence are not degraded through COP1 activity, leading to their accumulation and overabundance in the disease state [73]. Moreover, PCa samples lacking ETV1 translocations but harbouring *COP1* deletions displayed higher ETV1 protein levels, implicating *COP1* loss as a potential route to oncogenesis [73].

COP1 appears to act as a tumour suppressor through the regulation of PEA3 member protein levels in various forms of cancer. Over-expression of COP1 in the TNBC cell line MDA-MB-231 reduced migration and invasiveness, which could be recovered with concomitant ETV1 expression [74]. Analysis of TNBC tissues found that ETV1 protein levels were inversely correlated to those of COP1, and ETV1-positive tumours were associated with shorter survival times compared to patients with ETV1 negative tumours, whereas COP1 positive tumours gave significantly improved outcomes against COP1 negative tumours [74].

Transcriptional outputs arising from elevated ERK activity seen in *BRAF* V600-mutant melanomas and *KIT*-mutant gastrointestinal stromal tumours (GISTs) may also be mediated through ETV1 unconstrained by COP1. COP1 loss in A375 melanoma and GIST-T1 cell line-derived xenograft models led to increased ETV1 (and ETV4/5) levels and resistance to MAPK inhibitor treatments [75]. De novo activity-attenuating DET1 mutations were identified in two melanoma patients after vemurafenib (RAF inhibitor) treatment, further implicating COP1/DET1 loss as a driver of MAPK inhibitor resistance [75].

The relationship between COP1 and PEA3 members was also found to be important in regulating insulin secretion from pancreatic β cells, whereby hypoglycaemia caused by loss of Cop1 could be rescued by deletion of PEA3 family members in a mouse model [76]. The COP1-ETV axis is also active in neurons, as mice with *Cop1* deleted in neural stem cells exhibited an increase in Etv1, Etv4 and Etv5 expression and died soon after birth. This could be attributed directly to PEA3 family overabundance as lethality was reduced in *Cop1*-deleted mice lacking *Etv5* and heterozygous for *Etv1* [77]. Furthermore, lung branching defects in mice caused by prenatal *Cop1* gene inactivation could be partially rescued through introduction of *Etv5* loss-of-function mutant alleles, implicating COP1 control over ETV5 protein levels in lung developmental processes [78].

In summary, COP1-mediated ubiquitination of PEA3 members appears to be regulated by MAPK phosphorylation and the compensatory effects of mutations in genes of COP1 subunits and PEA3 members highlight the importance of this regulatory axis in several developmental contexts, including neurogenesis.

## 5. TCFs Showcase Mono-Ubiquitination as a Non-Destructive Mode of Repression

The ternary complex factor (TCF) subfamily of ETS transcription factors comprises ELK-1 and two additional members, ELK-3 (NET) and ELK-4 (SAP1). The name derives from the ternary complex that TCFs form with Serum Response Factor (SRF) at Serum Response Elements (SRE) in immediate early gene (IEG) targets (e.g., *CFOS*, *EGR1*) following phosphorylation of the C-terminal TAD by MAPKs [79,80,81]. The importance of TCFs in developmental processes is best highlighted by the disruption of mesoderm formation following Elk-1 depletion from *Xenopus* embryos, coupled with evidence pointing to TCF functionality emerging alongside mesoderm [82,83].

It was initially found that recombinant ELK-1 was readily polyubiquitinated in an in vitro ubiquitination assay using rabbit reticulocyte lysates [84]. Subsequently, ELK-1 was shown to be polyubiquitinated in cellulo with evidence of K48-linkages, albeit with a marginal impact on ELK-1 stability [85,86]. In contrast, a truncated version of ELK-1 (sELK-1—missing the N-terminal 54 residues) is far less stable than full-length ELK-1 due to a cryptic degron (CD—aas 167–196, Figure 1a) that in the absence of a proposed dimerisation interface (aas 7–32) promoted the rapid turnover of dimerisation-defective ELK-1 [85].

The only protein so far identified as a potential E3 ligase for TCFs is the F-box protein FBXO25, which complexes with SKP1, CUL1 and ROC1 to form an active RING E3 ligase [87]. FBXO25 was reported to interact with ELK-1 and promote its polyubiquitination and degradation by the 26S proteasome, thereby impairing ELK-1 target gene transcription [88]. However, although subsequent, independent work confirmed that FBXO25 interacted with ELK-1 in a region including the CD, its proposed impact on ELK-1 ubiquitination or transcriptional activity could not be substantiated [89].

In apparent contrast to other ETS proteins, TCFs may not be regulated mainly by polyubiquitination and turnover. The modified form of ELK-1 predominant in unstimulated cells is mono-ubiquitinated in the N-terminal ETS domain. Mutational analysis and MS revealed K35 as the primary modification site with ubiquitination also occurring at K52 and K59 [86]. Mono-ubiquitin conjugation decreased ELK-1 DNA binding and was reversed following mitogen stimulation of cells and consequent ELK-1 phosphorylation and activation. These observations, coupled with the increased transcriptional activity of a hypo-ubiquitinated ELK-1 mutant compared with wild-type ELK-1, suggest that ETS domain mono-ubiquitination is a repressive mark [86].

The main enzyme responsible for removing ubiquitin from ELK-1 appears to be USP17 (DUB3). The USP17 family of DUBs comprises a number of very similar proteins originating from a cell-cycle regulated multicopy gene that has oncogene character in a range of cancer pathologies, albeit with noted exceptions [90]. USP17 was found to interact with ELK-1 in a region encompassing the B-domain and CD (aas 93–189) and efficiently deubiquitinate both mono- and polyubiquitinated ELK-1, thereby augmenting its transcriptional activity, driving target gene expression and cell cycle entry [86,89]. Moreover, expression of a hypo-ubiquitinated ELK-1 mutant, but not wild-type ELK-1, partially rescued the proliferation defect caused by USP17 depletion in HEK293T cells, illustrating the contribution of this mechanism towards mitogen signalling [86]. The established role for ELK-1 as an AR tethering element suggests that further studies are warranted to decipher whether USP17 acts on ELK-1 to promote tumorigenesis in PCa [91,92,93].

Evidence for the polyubiquitination of ELK-3 and ELK-4 is sparce. ELK-3 has been shown to be polyubiquitinated in murine endothelial cells subjected to hypoxia [94]. More recently, ELK-4 was reported to interact directly through its C-terminal region with the adapter protein downstream of kinase 4 (DOK-4), which promoted its translocation to the cytoplasm and turnover, the latter being prevented with concomitant proteasomal inhibition [95]. Multiple lysine substitutions at predicted ubiquitin acceptor residues stabilised ELK-4 in the presence of DOK-4, further implying that DOK-4 control over ELK-4 protein levels occurs via the UPS. Accordingly, DOK-4 depletion in MDCK II kidney cells (where ELK-4 is the predominant TCF) stabilised ELK-4 and increased IEG expression and cell proliferation [95]. We have confirmed that ELK-3 and ELK-4 are ubiquitinated in cellulo and, when ubiquitinated, both serve as substrates for USP17 (our unpublished data).

In summary, ELK-1 exemplifies how mono-ubiquitination of ETS domain lysines can interfere reversibly with DNA binding to dampen transcriptional activity of TCFs, a mechanism hitherto unrecognised in other ETS proteins.

## 6. TEL and the PNT Domain as Drivers of Turnover or Fusion Protein Activity

The closely related transcriptional repressors ETV6 (TEL1—translocating E26 transforming-specific leukaemia 1) and ETV7 (TEL2) have functional roles in haematopoiesis and vascular development [96]. Both proteins contain N-terminal PNT domains that promote homo- and hetero-oligomerisation and influence the repression of target genes [97].

The PNT domain also participates in the polyubiquitination of ETV6 and ETV7 by an SCF complex containing the F-box and leucine rich repeat protein 6 (FBXL6), which interacts directly with both proteins through their PNT domains to promote proteasomal degradation [98]. This is also the case for Yan, the invertebrate orthologue of ETV6, although the mechanism does not appear to extend to other human PNT domain-containing ETS proteins, as FBXL6 was not able to bind to ETS-1 or ETS-2 [98]. This mode of recognition may also be subject to regulation, based on a previous finding suggesting that PIAS3-mediated SUMOylation of ETV6 monomers, but not oligomers formed through the PNT domain, were sensitized for UPS processing [99]. More recently, the sea urchin orthologue Yan/Tel was found to be phosphorylated by GSK-3β to promote its proteasomal degradation through recognition by the E3 ligase FBXW1A (β-TRCP) [100].

Several oncogenic ETV6 fusions have been described that differ significantly from the *TMPRSS2:ETS* fusions outlined above. Commonly, they express proteins in which the N-terminal PNT domain of ETV6 is fused to a tyrosine kinase domain, whereby the PNT domain promotes oligomerisation that leads to constitutive tyrosine kinase activity. One such example are gene rearrangements that fuse ETV6 to the tyrosine kinase domain of neurotrophic receptor tyrosine kinase 3 (NTRK3), forming a chimaeric oncoprotein known as EN, which is expressed in various cancers [101]. EN was shown to be polyubi-quitinated by ring finger protein 123 (RNF123, or KPC1), promoting its turnover by the proteasome [102]. Stimulation of insulin-like growth factor 1 receptor (IGF1R) promoted EN tyrosine phosphorylation and protected EN from RNF123 interaction, thus stabilising EN. This represents a potential therapeutic target, as IGF1R inhibitor treatment in EN-expressing EpH4 mouse mammary epithelial cell tumour-bearing mice destabilised EN and reduced cell proliferation [102].

Whether RNF123 interacts with the ETV6 or NTRK3 moiety of the fusion protein, and which region harbours lysine(s) targeted for modification are unknown, so it remains unclear whether other ETV6-fusions, or indeed ETV6 itself, are targeted similarly. However, another oncogenic ETV6 PNT-tyrosine kinase fusion occurs with the JAK2 JH1 domain, which is also regulated by the proteasome. In this case, suppressor of cytokine signalling 1 (SOCS1) was shown to interact with CUL-2 and promote ETV6-JAK2 polyubiquitination, which occurred through the JH1 domain independently of the ETV6 portion of the protein [103].

Building on the example of the ERGs, TEL proteins further exemplify how UPS regulation is altered in gene fusion products, with loss and/or gain of degrons and modification sites influencing the activity of these chimaeric proteins. Furthermore, TELs highlight that the conserved PNT domain can participate in ETS family member ubiquitination.

## 7. Discussion

Of the 28 human *ETS* genes, over half express proteins that have been found to be ubiquitinated and several of the E3 ligases responsible have been identified (Table 1). The reported consequences of their modification are destruction following addition of K48-linked polyubiquitin chains, or interference with DNA binding and nuclear export following mono-ubiquitination, both of which suppress ETS protein function, i.e., regulation of gene expression and concomitant impact on cell proliferation, differentiation and oncogenesis. Conceivably, mono-ubiquitination serves as an initiation event for polyubiquitination and turnover as well as impairing DNA binding and/or promoting nuclear export, although available data appear to rule out this possibility in the case of ELK-1 [86].

The highly conserved ETS domain confers on all family members the ability to bind to a short, conserved, purine-rich DNA motif, for which 4 distinct binding profiles have been characterised [104]. This finding implied significant overlap in target gene sets for different ETS proteins and accordingly, all ETS factors linked to cancer share the same binding profile [104]. In general E3 ligases that target these factors have TS character, notably SPOP and FBXW7, whereas their DUBs tend to promote ETS activity and cell proliferation, for example USP9X and USP17 [30,47,52,53,57,86]. This assignment is borne out by the action of these E3 ligases and DUBs on other known clients, although interestingly, the actions of USP17 appear to be ambiguous [90].

**Table 1:** E3 ligases and DUBs reported to facilitate ubiquitination and deubiquitination of human ETS proteins and their oncogenic fusions (* Disputed, ^Lacks ETS domain).

**ETS Protein**	**E3 Ligase**	**DUB**	**References**
ETS-1	COP1	USP9X	[30,35]
ETS-2	COP1	-	[35,40]
ERG	TRIM25, SPOP, FBXW7	USP9X	[47,49,52,53,57]
ETV1/ER81	COP1	-	[72,73]
ETV4/PEA3/E1AF	COP1	-	[72,73]
ETV5/ERM	COP1	-	[72,73]
ETV6/TEL1	FBXL6	-	[98]
ETV7/TEL2	FBXL6	-	[98]
ELF3/ESE1	FBXW1A	-	[105]
ELF4/MEF	SKP2	-	[106]
PU.1/SPI-1	FBXW7	USP22	[63,65]
ELK-1	FBXO25 *	USP17	[86,88,89]
**ETS Fusion**	**E3 Ligase**	**DUB**	**References**
TMPRSS2-ERG	TRIM25, FBXW7	USP9X	[47,49,57]
EWS-FLI-1/ERGB	-	USP19	[61]
ETV6/TEL1^-NTRK3	RNF123	-	[102]
ETV6/TEL1^-JAK2	SOCS1	-	[103]

The above rule has two exceptions. PU.1 is notable because within the haematopoietic lineage it drives differentiation of precursor cells into monocytes and macrophages [62]. In this instance, inhibition of FBXW7 or enhancement of USP22 activity towards PU.1 promoted precursor differentiation [63,65]. The other exception is the SAM pointed domain-containing ETS transcription factor (SPDEF), the only member of the PDEF subfamily, whose anti-metastatic and anti-tumorigenic activities in PCa and hepatocellular carcinoma cells respectively are antagonised by UPS processing following phosphorylation by CDK11B [107,108]. In these situations, SPDEF has TS character, whereas CDK11B and the as yet unidentified downstream E3 ligase(s) have oncogenic properties.

The best-studied sites of ubiquitin conjugation within ETS proteins lie predominantly within the well-structured ETS domain and include highly conserved lysines on the β2/β3 strands and α3 helix that participate in DNA-binding [86] (Table 2). It follows that ubiquitin conjugated to these sites, notably α3 residues K388/K334/K59 in ETS-1/FLI-1/ELK-1 respectively (Figure 1b,c), would be incompatible with DNA-binding and target gene regulation. Although the data obtained from large scale proteomic studies could be skewed by coverage issues, this pattern appears distinct from that of phosphorylation, which is frequently targeted to unstructured regions. Conceivably, the constraints of isopeptide bond formation favour lysines within stable secondary structures. However, degrons or E3 recognition motifs are commonly distal to target lysine(s), may lie within unstructured regions, including TADs, and be subject to modulatory phosphorylation, although in the case of ETV4, the C-terminal region encompassing the ETS domain appears to be essential for turnover [68].

Diversity is a hallmark of ETS proteins and in the case of ubiquitination the separation of substrate recognition and modification may have facilitated regulatory diversification. This process is acutely illustrated with fusion proteins: for example, *TMPRSS2:ERG* and *TMPRSS2:ETV1* fusion products escape SPOP and COP1-mediated ubiquitination respectively through loss of N-terminal recognition motifs [52,53,73]. Conversely, to stabilise EWS-FLI-1 fusions USP19 binds within N-terminal EWS sequences and deubiquitinates the C-terminal FLI-1 ETS domain [61]. Conceivably, regulatory diversification may also occur between splice variants of the same protein that retain or exclude degron motifs.

The preference for conserved lysines within the ETS domain could be interpreted as evidence for mechanistic or pathway conservation among ETS proteins. Consistent with this notion are the multiple substrates observed for COP1 (ETS1/2, ETV1/4/5), FBXW7 (ERG, PU.1), USP9X (ETS1, ERG) and USP17 (ELK-1/3/4). But again, the evidence for divergence is far more compelling, with ubiquitination of 11 ETS proteins attributed to 7 distinct E3 ligase complexes (Table 1). Furthermore, while ERG is a substrate for both SPOP and FBXW7, its closest relative FLI-1 is likely a substrate for neither as it lacks the SBC and CPD motifs shown to be active in ERG [52,53,57].

Another hallmark of ETS proteins is their propensity for regulation by phosphorylation, which extends from their activation, well-illustrated by TCFs, to their turnover. A prime example here is ERG, with two phosphodegrons that undergo priming phosphorylation by protein kinases (CK1δ and WEE1) activated in response to DNA damage [52,57]. Rapid turnover of E74-like factor 4 (ELF4/MEF, of the ELF subfamily) after G1/S transition by F-box ligase SKP2 is triggered by C-terminal phosphorylation by CDK2-Cyclin A1 [106]. Phosphorylation can also protect ETS proteins from degradation. SRC kinases are able to phosphorylate a tyrosine residue adjacent to the COP in ETS-1 to inhibit COP1 binding [35]. Another example is ELF3 (ESE1, of the ESE subfamily) a labile protein readily degraded following polyubiquitination by FBXW1A (β-TrCP1) and protection is afforded through phosphorylation by p21-activated kinase 1 (PAK1) at S207 in a serine and aspartic acid-rich (SAR) domain. Stabilisation of ELF3 by PAK1 promoted ERK signalling and anchorage-independent growth in breast cancer cells [105].

In conclusion, ETS factors provide informative insights in gene diversification at multiple levels, including their interrelationships with the UPS. Although ubiquitination of the ETS domain is a common feature, the enzymes that determine ubiquitin status are diverse and selective, based on recognition of distal degrons or binding motifs, of which several are also targets of regulatory phosphorylation. Analysis of *ets* gene mRNA levels in human cells and tissues detected co-expression of multiple paralogues, implying scope for the cell-specific regulation of ETS protein levels by the UPS [117]. Similarly, most cells are unlikely to express a full complement of the ubiquitination machinery.

E3 ligase complexes that target ETS proteins linked to oncogenesis exercise tumour suppression and these ETS proteins exhibit a range of evasive strategies, including gene fusion-mediated loss of degron sequences, E3 ligase inactivation by point mutation and up-regulation of deubiquitinases, that define multiple cancer types and offer new therapeutic targets. Despite recent progress, there is much more to be learned about ubiquitin-mediated control of ETS factors.

## Figures and Tables

**Table 2:** ETS-domain lysines identified as ubiquitination sites from targeted and large-scale proteomic datasets and/or mutational analysis (human-produced with use of PhosphoSitePlus). Highlighted in red is the conserved α3 helix lysine found to be a target in multiple ETS proteins.

ETS Protein	ETS Domain Ubiquitination Site	References
ETS-1	K377, K388, K399, K404	[30,109,110,111,112]
ETS-2	K416, K427, K432	[109,110,111,112]
FLI-1/ERGB	K334 (K380 EWS-FLI)	[60,112]
ETV2/ER71	K294	[109,110,112]
ETV6/TEL1	K393, K403	[112,113]
ETV7/TEL2	K293	[114]
GABPα	K359, K366, K373	[109,110,112,113,114,115,116]
ELF1	K226, K244	[109,110,112]
ELF2/NERF	K290	[110]
ELF3/ESE1	K294, K328	[115]
ELK-1	K35, K52, K59	[86]
ELK-3/NET	K83	[110]

## Data Availability

Not applicable.

## References

[1] Lemon B., Tjian R. (2000). Orchestrated response: A symphony of transcription factors for gene control. Genes Dev..

[2] Karim F.D., Urness L.D., Thummel C.S., Klemsz M.J., McKercher S.R. (1990). The ETS-domaln: A new DNA-binding motif that recognizes a purine-rich core DNA sequence. Genes Dev..

[3] Leprince D., Gegonne A., Coll J., de Taisne C., Schneeberger A., Lagrou C., Stehelin D. (1983). A putative second cell-derived oncogene of the avian leukaemia retrovirus E26. Nature.

[4] Janknecht R., Nordheim A. (1993). Gene regulation by Ets proteins. Biochim. Biophys. Acta BBA—Rev. Cancer.

[5] Sizemore G.M., Pitarresi J.R., Balakrishnan S., Ostrowski M.C. (2017). The ETS family of oncogenic transcription factors in solid tumours. Nat. Rev. Cancer.

[6] Hollenhorst P.C., McIntosh L.P., Graves B.J. (2011). Genomic and biochemical insights into the specificity of ETS transcription factors. Annu. Rev. Biochem..

[7] Mackereth C.D., Schärpf M., Gentile L.N., MacIntosh S.E., Slupsky C.M., McIntosh L.P. (2004). Diversity in structure and function of the Ets family PNT domains. J. Mol. Biol..

[8] Selvaraj N., Kedage V., Hollenhorst P.C. (2015). Comparison of MAPK specificity across the ETS transcription factor family identifies a high-affinity ERK interaction required for ERG function in prostate cells. Cell Commun. Signal..

[9] Maroulakou I.G., Bowe D.B. (2000). Expression and function of Ets transcription factors in mammalian development: A regulatory network. Oncogene.

[10] Hollenhorst P.C. (2012). RAS/ERK pathway transcriptional regulation through ETS/AP-1 binding sites. Small GTPases.

[11] Liu M., Gao W., Van Velkinburgh J.C., Wu Y., Ni B., Tian Y. (2016). Role of Ets Proteins in Development, Differentiation, and Function of T-Cell Subsets. Med. Res. Rev..

[12] Fry E.A., Mallakin A., Inoue K. (2018). Translocations involving ETS family proteins in human cancer. Integr. Cancer Sci. Ther..

[13] Hsing M., Wang Y., Rennie P.S., Cox M.E., Cherkasov A. (2020). ETS transcription factors as emerging drug targets in cancer. Med. Res. Rev..

[14] Yang S.-H., Jaffray E., Hay R.T., Sharrocks A.D. (2003). Dynamic interplay of the SUMO and ERK pathways in regulating Elk-1 transcriptional activity. Mol. Cell.

[15] Guo B., Panagiotaki N., Warwood S., Sharrocks A.D. (2011). Dynamic modification of the ETS transcription factor PEA3 by sumoylation and p300-mediated acetylation. Nucleic Acids Res..

[16] McClellan A.J., Laugesen S.H., Ellgaard L. (2019). Cellular functions and molecular mechanisms of non-lysine ubiquitination. Open Biol..

[17] Stewart M.D., Ritterhoff T., Klevit R.E., Brzovic P.S. (2016). E2 enzymes: More than just middle men. Cell Res..

[18] Mevissen T.E.T., Komander D. (2017). Mechanisms of deubiquitinase specificity and regulation. Annu. Rev. Biochem..

[19] Zheng N., Shabek N. (2017). Ubiquitin ligases: Structure, function, and regulation. Annu. Rev. Biochem..

[20] George A.J., Hoffiz Y.C., Charles A.J., Zhu Y., Mabb A.M. (2018). A comprehensive atlas of E3 ubiquitin ligase mutations in neurological disorders. Front. Genet..

[21] Yau R., Rape M. (2016). The increasing complexity of the ubiquitin code. Nat. Cell Biol..

[22] Tracz M., Bialek W. (2021). Beyond K48 and K63: Non-canonical protein ubiquitination. Cell. Mol. Biol. Lett..

[23] Yuan T., Yan F., Ying M., Cao J., He Q., Zhu H., Yang B. (2018). Inhibition of Ubiquitin-Specific Proteases as a Novel Anticancer Therapeutic Strategy. Front. Pharmacol..

[24] Bulatov E., Zagidullin A., Valiullina A., Sayarova R., Rizvanov A. (2018). Small molecule modulators of RING-type E3 ligases: MDM and cullin families as targets. Front. Pharmacol..

[25] Garrett-Sinha L.A. (2013). Review of Ets1 structure, function, and roles in immunity. Cell. Mol. Life Sci..

[26] Lie-Venema H., Gittenberger-De Groot A.C., Van Empel L.J.P., Boot M.J., Kerkdijk H., De Kant E., DeRuiter M.C. (2003). Ets-1 and Ets-2 transcription factors are essential for normal coronary and myocardial development in chicken embryos. Circ. Res..

[27] Nie S., Bronner M.E. (2015). Dual developmental role of transcriptional regulator Ets1 in Xenopus cardiac neural crest vs. heart mesoderm. Cardiovasc. Res..

[28] Taveirne S., Wahlen S., van Loocke W., Kiekens L., Persyn E., van Ammel E., de Mulder K., Roels J., Tilleman L., Aumercier M. (2020). The transcription factor ETS1 is an important regulator of human NK cell development and terminal differentiation. Blood.

[29] Ji Z., Degerny C., Vintonenko N., Deheuninck J., Foveau B., Leroy C., Coll J., Tulasne D., Baert J.L., Fafeur V. (2007). Regulation of the Ets-1 transcription factor by sumoylation and ubiquitinylation. Oncogene.

[30] Potu H., Peterson L.F., Kandarpa M., Pal A., Sun H., Durham A., Harms P.W., Hollenhorst P.C., Eskiocak U., Talpaz M. (2017). Usp9x regulates Ets-1 ubiquitination and stability to control NRAS expression and tumorigenicity in melanoma. Nat. Commun..

[31] Ohtake F., Tsuchiya H., Saeki Y., Tanaka K. (2018). K63 ubiquitylation triggers proteasomal degradation by seeding branched ubiquitin chains. Proc. Natl. Acad. Sci. USA.

[32] Fulzele A., Bennett E.J. (2018). Ubiquitin diGLY proteomics as an approach to identify and quantify the ubiquitin-modified proteome. Methods Mol. Biol..

[33] Mo Y., Vaessen B., Johnston K. (2000). Structure of the Elk-1–DNA complex reveals how DNA- distal residues affect ETS domain recognition of DNA. Nat. Struct. Biol..

[34] Marine J.C. (2012). Spotlight on the role of COP1 in tumorigenesis. Nat. Rev. Cancer.

[35] Lu G., Zhang Q., Huang Y., Song J., Tomaino R., Ehrenberger T., Lim E., Liu W., Bronson R.T., Bowden M. (2014). Phosphorylation of ETS1 by src family kinases prevents its recognition by the COP1 tumor suppressor. Cancer Cell.

[36] Emanuele M.J., Elia A.E.H., Xu Q., Thoma C.R., Izhar L., Leng Y., Guo A., Chen Y.N., Rush J., Hsu P.W.C. (2011). Global identification of modular cullin-RING ligase substrates. Cell.

[37] Yu J.C., Chen J.-R., Lin C.-H., Zhang G., Lam P.-S., Wenger K.H., Mozaffari F.B., Huang S.-T., Borke J.L. (2009). Tensile Strain-Induced Ets-2 Phosphorylation by CaMKII and the Homeostasis of Cranial Sutures. Plast. Reconstr. Surg..

[38] Nishida T., Terashima M., Fukami K., Yamada Y. (2007). PIASy controls ubiquitination-dependent proteasomal degradation of Ets-1. Biochem. J..

[39] Guen V.J., Gamble C., Flajolet M., Unger S., Thollet A., Ferandin Y., Superti-Furga A., Cohen P.A., Meijer L., Colas P. (2013). CDK10/cyclin M is a protein kinase that controls ETS2 degradation and is deficient in STAR syndrome. Proc. Natl. Acad. Sci. USA.

[40] Carrero Z.I., Kollareddy M., Chauhan K.M., Ramakrishnan G., Martinez L.A. (2016). Mutant p53 protects ETS2 from non-canonical COP1/DET1 dependent degradation. Oncotarget.

[41] Bargonetti J., Prives C. (2019). Gain-of-function mutant p53: History and speculation. J. Mol. Cell Biol..

[42] Li M., Shin Y.H., Hou L., Huang X., Wei Z., Klann E., Zhang P. (2008). The adaptor protein of the anaphase promoting complex Cdh1 is essential in maintaining replicative lifespan and in learning and memory. Nat. Cell Biol..

[43] Murtaza M., Jolly L.A., Gecz J., Wood S.A. (2015). La FAM fatale: USP9X in development and disease. Cell. Mol. Life Sci..

[44] Knudsen K.J., Rehn M., Hasemann M.S., Rapin N., Bagger F.O., Ohlsson E., Willer A., Frank A.K., Søndergaard E., Jendholm J. (2015). ERG promotes the maintenance of hematopoietic stem cells by restricting their differentiation. Genes Dev..

[45] Spyropoulos D.D., Pharr P.N., Lavenburg K.R., Jackers P., Papas T.S., Ogawa M., Watson D.K. (2000). Hemorrhage, Impaired Hematopoiesis, and Lethality in Mouse Embryos Carrying a Targeted Disruption of the Fli1Transcription Factor. Mol. Cell. Biol..

[46] Tomlins S.A., Rhodes D.R., Perner S., Dhanasekaran S.M., Mehra R., Sun X.W., Varambally S., Cao X., Tchinda J., Kuefer R. (2005). Recurrent fusion of TMPRSS2 and ETS transcription factor genes in prostate cancer. Science.

[47] Wang S., Kollipara R.K., Srivastava N., Li R., Ravindranathan P., Hernandez E., Freeman E., Humphries C.G., Kapur P., Lotan Y. (2014). Ablation of the oncogenic transcription factor ERG by deubiquitinase inhibition in prostate cancer. Proc. Natl. Acad. Sci. USA.

[48] Martín-Vicente M., Medrano L.M., Resino S., García-Sastre A., Martínez I. (2017). TRIM25 in the regulation of the antiviral innate immunity. Front. Immunol..

[49] Wang S., Kollipara R.K., Humphries C.G., Ma S.H., Hutchinson R., Li R., Siddiqui J., Tomlins S.A., Raj G.V., Kittler R. (2016). The ubiquitin ligase TRIM25 targets ERG for degradation in prostate cancer. Oncotarget.

[50] Cheng J., Guo J., Wang Z., North B.J., Tao K., Dai X., Wei W. (2018). Functional analysis of Cullin 3 E3 ligases in tumorigenesis. Biochim. Biophys. Acta BBA—Rev. Cancer.

[51] Barbieri C.E., Baca S.C., Lawrence M.S., Demichelis F., Blattner M., Theurillat J.P., White T.A., Stojanov P., Van Allen E., Stransky N. (2012). Exome sequencing identifies recurrent SPOP, FOXA1 and MED12 mutations in prostate cancer. Nat. Genet..

[52] An J., Ren S., Murphy S.J., Dalangood S., Chang C., Pang X., Cui Y., Wang L., Pan Y., Zhang X. (2015). Truncated ERG Oncoproteins from TMPRSS2-ERG Fusions Are Resistant to SPOP-Mediated Proteasome Degradation. Mol. Cell.

[53] Gan W., Dai X., Lunardi A., Li Z., Inuzuka H., Liu P., Varmeh S., Zhang J., Cheng L., Sun Y. (2015). SPOP Promotes Ubiquitination and Degradation of the ERG Oncoprotein to Suppress Prostate Cancer Progression. Mol. Cell.

[54] Shoag J., Liu D., Blattner M., Sboner A., Park K., Deonarine L., Robinson B.D., Mosquera J.M., Chen Y., Rubin M.A. (2018). SPOP mutation drives prostate neoplasia without stabilizing oncogenic transcription factor ERG. J. Clin. Investig..

[55] Abeshouse A., Ahn J., Akbani R., Ally A., Amin S., Andry C.D., Annala M., Aprikian A., Armenia J., Arora A. (2015). The Molecular Taxonomy of Primary Prostate Cancer. Cell.

[56] Yeh C.H., Bellon M., Nicot C. (2018). FBXW7: A critical tumor suppressor of human cancers. Mol. Cancer.

[57] Hong Z., Zhang W., Ding D., Huang Z., Yan Y., Cao W., Pan Y., Hou X., Weroha S.J., Karnes R.J. (2020). DNA Damage Promotes TMPRSS2-ERG Oncoprotein Destruction and Prostate Cancer Suppression via Signaling Converged by GSK3β and WEE1. Mol. Cell.

[58] Hermida M.A., Dinesh Kumar J., Leslie N.R. (2017). GSK3 and its interactions with the PI3K/AKT/mTOR signalling network. Adv. Biol. Regul..

[59] Delattre O., Zucman J., Plougastel B., Desmaze C., Melot T., Peter M., Kovar H., Joubert I., De Jong P., Rouleau G. (1992). Gene fusion with an ETS DNA-binding domain caused by chromosome translocation in human tumours. Nature.

[60] Gierisch M.E., Pfistner F., Lopez-Garcia L.A., Harder L., Schäfer B.W., Niggli F.K. (2016). Proteasomal degradation of the EWS-FLI1 fusion protein is regulated by a single lysine residue. J. Biol. Chem..

[61] Gierisch M.E., Pedot G., Walser F., Lopez-Garcia L.A., Jaaks P., Niggli F.K., Schäfer B.W. (2019). USP19 deubiquitinates EWS-FLI1 to regulate Ewing sarcoma growth. Sci. Rep..

[62] Metcalf D., Dakic A., Mifsud S., Di Rago L., Wu L., Nutt S. (2006). Inactivation of PU.1 in adult mice leads to the development of myeloid leukemia. Proc. Natl. Acad. Sci. USA.

[63] Mishra M., Thacker G., Sharma A., Singh A.K., Upadhyay V., Sanyal S., Verma S.P., Tripathi A.K., Bhatt M.L.B., Trivedi A.K. (2021). FBW7 Inhibits Myeloid Differentiation in Acute Myeloid Leukemia via GSK3-Dependent Ubiquitination of PU.1. Mol. Cancer Res..

[64] Zhao Y., Lang G., Ito S., Bonnet J., Metzger E., Sawatsubashi S., Suzuki E., Le Guezennec X., Stunnenberg H.G., Krasnov A. (2008). A TFTC/STAGA Module Mediates Histone H2A and H2B Deubiquitination, Coactivates Nuclear Receptors, and Counteracts Heterochromatin Silencing. Mol. Cell.

[65] Melo-Cardenas J., Xu Y., Wei J., Tan C., Kong S., Gao B., Montauti E., Kirsammer G., Licht J.D., Yu J. (2018). USP22 deficiency leads to myeloid leukemia upon oncogenic Kras activation through a PU.1-dependent mechanism. Blood.

[66] Oh S., Shin S., Janknecht R. (2012). ETV1, 4 and 5: An oncogenic subfamily of ETS transcription factors. Biochim. Biophys. Acta BBA—Rev. Cancer.

[67] Herriges J.C., Verheyden J.M., Zhang Z., Sui P., Zhang Y., Anderson M.J., Swing D.A., Zhang Y., Lewandoski M., Sun X. (2015). FGF-Regulated ETV Transcription Factors Control FGF-SHH Feedback Loop in Lung Branching. Dev. Cell.

[68] Takahashi A., Higashino F., Aoyagi M., Yoshida K., Itoh M., Kobayashi M., Totsuka Y., Kohgo T., Shindoh M. (2005). E1AF degradation by a ubiquitin-proteasome pathway. Biochem. Biophys. Res. Commun..

[69] Guo B., Sharrocks A.D. (2009). Extracellular Signal-Regulated Kinase Mitogen-Activated Protein Kinase Signaling Initiates a Dynamic Interplay between Sumoylation and Ubiquitination To Regulate the Activity of the Transcriptional Activator PEA3. Mol. Cell. Biol..

[70] Xiao J., Yang S., Shen P., Wang Y., Sun H., Ji F., Zhou D. (2017). Phosphorylation of ETV4 at Ser73 by ERK kinase could block ETV4 ubiquitination degradation in colorectal cancer. Biochem. Biophys. Res. Commun..

[71] Baert J.L., Beaudoin C., Monte D., Degerny C., Mauen S., De Launoit Y. (2007). The 26S proteasome system degrades the ERM transcription factor and regulates its transcription-enhancing activity. Oncogene.

[72] Baert J.L., Monte D., Verreman K., Degerny C., Coutte L., De Launoit Y. (2010). The E3 ubiquitin ligase complex component COP1 regulates PEA3 group member stability and transcriptional activity. Oncogene.

[73] Vitari A.C., Leong K.G., Newton K., Yee C., O’Rourke K., Liu J., Phu L., Vij R., Ferrando R., Couto S.S. (2011). COP1 is a tumour suppressor that causes degradation of ETS transcription factors. Nature.

[74] Ouyang M., Wang H., Ma J., Lü W., Li J., Yao C., Chang G., Bi J., Wang S., Wang W. (2015). COP1, the negative regulator of ETV1, influences prognosis in triple-negative breast cancer. BMC Cancer.

[75] Xie Y., Cao Z., Wong E.W.P., Guan Y., Ma W., Zhang J.Q., Walczak E.G., Murphy D., Ran L., Sirota I. (2018). COP1/DET1/ETS axis regulates ERK transcriptome and sensitivity to MAPK inhibitors. J. Clin. Investig..

[76] Suriben R., Kaihara K.A., Paolino M., Reichelt M., Kummerfeld S.K., Modrusan Z., Dugger D.L., Newton K., Sagolla M., Webster J.D. (2015). β-Cell Insulin Secretion Requires the Ubiquitin Ligase COP1. Cell.

[77] Newton K., Dugger D.L., Sengupta-Ghosh A., Ferrando R.E., Chu F., Tao J., Lam W., Haller S., Chan S., Sa S. (2018). Ubiquitin ligase COP1 coordinates transcriptional programs that control cell type specification in the developing mouse brain. Proc. Natl. Acad. Sci. USA.

[78] Zhanga Y., Yokoyamaa S., Herrigesa J.C., Zhanga Z., Younga R.E., Verheydena J.M., Suna X. (2016). E3 ubiquitin ligase RFWD2 controls lung branching through protein-level regulation of ETV transcription factors. Proc. Natl. Acad. Sci. USA.

[79] Marais R., Wynne J., Treisman R. (1993). The SRF accessory protein Elk-1 contains a growth factor-regulated transcriptional activation domain. Cell.

[80] Janknecht R., Ernst W.H., Pingoud V., Nordheim A. (1993). Activation of ternary complex factor Elk-1 by MAP kinases. EMBO J..

[81] Gille H., Kortenjann M., Thomae O., Moomaw C., Slaughter C., Cobb M.H., Shaw P.E. (1995). ERK phosphorylation potentiates Elk-1-mediated ternary complex formation and transactivation. EMBO J..

[82] Nentwich O., Dingwell K.S., Nordheim A., Smith J.C. (2009). Downstream of FGF during mesoderm formation in Xenopus: The roles of Elk-1 and Egr-1. Dev. Biol..

[83] Saxton J., Ferjentsik Z., Ducker C., Johnson A.D., Shaw P.E. (2016). Stepwise evolution of Elk-1 in early deuterostomes. FEBS J..

[84] Fuchs S.Y., Xie B., Adler V., Fried V.A., Davis R.J., Ronai Z. (1997). C-jun NH2-terminal kinases target the ubiquitination of their associated transcription factors. J. Biol. Chem..

[85] Evans E.L., Saxton J., Shelton S.J., Begitt A., Holliday N.D., Hipskind R.A., Shaw P.E. (2011). Dimer formation and conformational flexibility ensure cytoplasmic stability and nuclear accumulation of Elk-1. Nucleic Acids Res..

[86] Ducker C., Chow L.K.Y., Saxton J., Handwerger J., McGregor A., Strahl T., Layfield R., Shaw P.E. (2019). De-ubiquitination of ELK-1 by USP17 potentiates mitogenic gene expression and cell proliferation. Nucleic Acids Res..

[87] Hagens O., Minina E., Schweiger S., Ropers H.H., Kalscheuer V. (2006). Characterization of FBX25, encoding a novel brain-expressed F-box protein. Biochim. Biophys. Acta BBA—Gen. Subj..

[88] Teixeira F.R., Manfiolli A.O., Soares C.S., Baqui M.M.A., Koide T., Gomes M.D. (2013). The F-box protein FBXO25 promotes the proteasome-dependent degradation of ELK-1 protein. J. Biol. Chem..

[89] Quintero-Barceinas R.S., Gehringer F., Ducker C., Saxton J., Shaw P.E. (2021). ELK-1 ubiquitination status and transcriptional activity are modulated independently of F-Box protein FBXO25. J. Biol. Chem..

[90] Ducker C., Shaw P.E. (2021). USP17-mediated de-ubiquitination and cancer: Clients cluster around the cell cycle. Int. J. Biochem. Cell Biol..

[91] Rosati R., Patki M., Chari V., Dakshnamurthy S., McFall T., Saxton J., Kidder B.L., Shaw P.E., Ratnam M. (2016). The amino-terminal domain of the androgen receptor co-opts extracellular signal-regulated kinase (ERK) docking sites in ELK1 protein to induce sustained gene activation that supports prostate cancer cell growth. J. Biol. Chem..

[92] Rosati R., Polin L., Ducker C., Li J., Bao X., Selvakumar D., Kim S., Xhabija B., Larsen M., McFall T. (2018). Strategy for tumor-selective disruption of androgen receptor function in the spectrum of prostate cancer. Clin. Cancer Res..

[93] Pardy L., Rosati R., Soave C., Huang Y., Kim S., Ratnam M. (2020). The ternary complex factor protein ELK1 is an independent prognosticator of disease recurrence in prostate cancer. Prostate.

[94] Gross C., Buchwalter G., Dubois-Pot H., Cler E., Zheng H., Wasylyk B. (2007). The Ternary Complex Factor Net Is Downregulated by Hypoxia and Regulates Hypoxia-Responsive Genes. Mol. Cell. Biol..

[95] Hooker E., Baldwin C., Roodman V., Batra A., Isa N.N., Takano T., Lemay S. (2017). Binding and inhibition of the ternary complex factor Elk-4/Sap1 by the adapter protein Dok-4. Biochem. J..

[96] Rasighaemi P., Ward A.C. (2017). ETV6 and ETV7: Siblings in hematopoiesis and its disruption in disease. Crit. Rev. Oncol. Hematol..

[97] Kim C.A., Phillips M.L., Kim W., Gingery M., Tran H.H., Robinson M.A., Faham S., Bowie J.U. (2001). Polymerization of the SAM domain of TEL in leukemogenesis and transcriptional repression. EMBO J..

[98] Roukens M.G., Alloul-Ramdhani M., Moghadasi S., Op den Brouw M., Baker D.A. (2008). Downregulation of Vertebrate Tel (ETV6) and Drosophila Yan Is Facilitated by an Evolutionarily Conserved Mechanism of F-Box-Mediated Ubiquitination. Mol. Cell. Biol..

[99] Roukens M.G., Alloul-Ramdhani M., Vertegaal A.C.O., Anvarian Z., Balog C.I.A., Deelder A.M., Hensbergen P.J., Baker D.A. (2008). Identification of a New Site of Sumoylation on Tel (ETV6) Uncovers a PIAS-Dependent Mode of Regulating Tel Function. Mol. Cell. Biol..

[100] Molina M.D., Quirin M., Haillot E., De Crozé N., Range R., Rouel M., Jimenez F., Amrouche R., Chessel A., Lepage T. (2018). MAPK and GSK3/ß-TRCP-mediated degradation of the maternal Ets domain transcriptional repressor Yan/Tel controls the spatial expression of nodal in the sea urchin embryo. PLoS Genet..

[101] Lannon C.L., Sorensen P.H.B. (2005). ETV6-NTRK3: A chimeric protein tyrosine kinase with transformation activity in multiple cell lineages. Semin. Cancer Biol..

[102] Tognon C.E., Rafn B., Cetinbas N.M., Kamura T., Trigo G., Rotblat B., Okumura F., Matsumoto M., Chow C., Davare M. (2018). Insulin-like growth factor 1 receptor stabilizes the ETV6-NTRK3 chimeric oncoprotein by blocking its KPC1/Rnf123-mediated proteasomal degradation. J. Biol. Chem..

[103] Kamizono S., Hanada T., Yasukawa H., Minoguchi S., Kato R., Minoguchi M., Hattori K., Hatakeyama S., Yada M., Morita S. (2001). The SOCS Box of SOCS-1 Accelerates Ubiquitin-dependent Proteolysis of TEL-JAK2. J. Biol. Chem..

[104] Wei G.H., Badis G., Berger M.F., Kivioja T., Palin K., Enge M., Bonke M., Jolma A., Varjosalo M., Gehrke A.R. (2010). Genome-wide analysis of ETS-family DNA-binding in vitro and in vivo. EMBO J..

[105] Manavathi B., Rayala S.K., Kumar R. (2007). Phosphorylation-dependent regulation of stability and transforming potential of ETS transcriptional factor ESE-1 by p21-activated kinase. J. Biol. Chem..

[106] Liu Y., Hedvat C.V., Mao S., Zhu X.-H., Yao J., Nguyen H., Koff A., Nimer S.D. (2006). The ETS Protein MEF Is Regulated by Phosphorylation-Dependent Proteolysis via the Protein-Ubiquitin Ligase SCFSkp2. Mol. Cell. Biol..

[107] Tamura R.E., Paccez J.D., Duncan K.C., Morale M.G., Simabuco F.M., Dillon S., Correa R.G., Gu X., Libermann T.A., Zerbini L.F. (2016). GADD45α and γ interaction with CDK11p58 regulates SPDEF protein stability and SPDEF-mediated effects on cancer cell migration. Oncotarget.

[108] Guo J.C., Yang Y.J., Guo M., Zhang J.Q., Zheng J.F., Liu Z. (2020). Involvement of CDK11B-mediated SPDEF ubiquitination and SPDEF-mediated microRNA-448 activation in the oncogenicity and self-renewal of hepatocellular carcinoma stem cells. Cancer Gene Ther..

[109] Mertins P., Qiao J.W., Patel J., Udeshi N.D., Clauser K.R., Mani D.R., Burgess M.W., Gillette M.A., Jaffe J.D., Carr S.A. (2013). Integrated proteomic analysis of post-translational modifications by serial enrichment. Nat. Methods.

[110] Udeshi N.D., Svinkina T., Mertins P., Kuhn E., Mani D.R., Qiao J.W., Carr S.A. (2013). Refined preparation and use of anti-diglycine remnant (K-ε-GG) antibody enables routine quantification of 10,000s of ubiquitination sites in single proteomics experiments. Mol. Cell. Proteom..

[111] Lumpkin R.J., Gu H., Zhu Y., Leonard M., Ahmad A.S., Clauser K.R., Meyer J.G., Bennett E.J., Komives E.A. (2017). Site-specific identification and quantitation of endogenous SUMO modifications under native conditions. Nat. Commun..

[112] Akimov V., Barrio-Hernandez I., Hansen S.V.F., Hallenborg P., Pedersen A.K., Bekker-Jensen D.B., Puglia M., Christensen S.D.K., Vanselow J.T., Nielsen M.M. (2018). Ubisite approach for comprehensive mapping of lysine and n-terminal ubiquitination sites. Nat. Struct. Mol. Biol..

[113] Povlsen L.K., Beli P., Wagner S.A., Poulsen S.L., Sylvestersen K.B., Poulsen J.W., Nielsen M.L., Bekker-Jensen S., Mailand N., Choudhary C. (2012). Systems-wide analysis of ubiquitylation dynamics reveals a key role for PAF15 ubiquitylation in DNA-damage bypass. Nat. Cell Biol..

[114] Wagner S.A., Beli P., Weinert B.T., Nielsen M.L., Cox J., Mann M., Choudhary C. (2011). A Proteome-wide, Quantitative Survey of In Vivo Ubiquitylation Sites Reveals Widespread Regulatory Roles. Mol. Cell. Proteom..

[115] Kim W., Bennett E.J., Huttlin E.L., Guo A., Li J., Possemato A., Sowa M.E., Rad R., Rush J., Comb M.J. (2011). Systematic and quantitative assessment of the ubiquitin-modified proteome. Mol. Cell.

[116] Boeing S., Williamson L., Encheva V., Gori I., Saunders R.E., Instrell R., Aygün O., Rodriguez-Martinez M., Weems J.C., Kelly G.P. (2016). Multiomic Analysis of the UV-Induced DNA Damage Response. Cell Rep..

[117] Hollenhorst P.C., Jones D.A., Graves B.J. (2004). Expression profiles frame the promoter specificity dilemma of the ETS family of transcription factors. Nucleic Acids Res..

